# Correction: Enríquez-Rodríguez et al. Metabolomic Signatures Predict Seven-Year Mortality in Clinically Stable COPD Patients. *Int. J. Mol. Sci.* 2025, *26*, 6373

**DOI:** 10.3390/ijms262010217

**Published:** 2025-10-21

**Authors:** César Jessé Enríquez-Rodríguez, Bella Agranovich, Sergi Pascual-Guàrdia, Rosa Faner, Ramon Camps-Ubach, Ady Castro-Acosta, José Luis López-Campos, Germán Peces-Barba, Luis Seijo, Oswaldo Antonio Caguana-Vélez, Diego Rodríguez-Chiaradia, Esther Barreiro, Eduard Monsó, Borja G. Cosío, Ifat Abramovich, Alvar Agustí, Carme Casadevall, Joaquim Gea, on behalf of the BIOMEPOC Group

**Affiliations:** 1 Hospital del Mar Research Institute, Servei de Pneumologia, Hospital del Mar, 08003 Barcelona, Spain; cesarjesse.enriquez01@alumni.upf.edu (C.J.E.-R.); spascual@hmar.cat (S.P.-G.); ramon.camps.ubach@hmar.cat (R.C.-U.); ocaguana@hmar.cat (O.A.C.-V.); darodriguez@hmar.cat (D.R.-C.); ebarreiro@researchmar.net (E.B.); carme.casadevall@upf.edu (C.C.); 2 MELIS Department, Universitat Pompeu Fabra, 08003 Barcelona, Spain; 3 Centro de Investigación Biomédica en Red, Área de Enfermedades Respiratorias (CIBERES), ISCiii, 28029 Madrid, Spain; rfaner@recerca.clinic.cat (R.F.); lcampos@separ.es (J.L.L.-C.); gpecesbarba@gmail.com (G.P.-B.); lseijo@unav.es (L.S.); eduardmonsomolas@gmail.com (E.M.); borja.cosio@ssib.es (B.G.C.); aagusti@clinic.cat (A.A.); 4 The Ruth and Bruce Rappaport Faculty of Medicine, Technion, Israel Institute of Technology, Haifa 3525433, Israel; bellakr@technion.ac.il (B.A.); ifat.a@technion.ac.il (I.A.); 5 Servei de Pneumologia (Institut Clínic de Respiratori), Hospital Clínic—Fundació Clínic per la Recerca Biomèdica, Universitat de Barcelona, 08036 Barcelona, Spain; 6 Servicio de Neumología, Hospital 12 de Octubre, 28041 Madrid, Spain; ady@h12o.es; 7 Unidad Médico-Quirúrgica de Enfermedades Respiratorias, Hospital Universitario Virgen del Rocío, Universidad de Sevilla, 41012 Sevilla, Spain; 8 Servicio de Neumología, Fundación Jiménez Díaz, Universidad Autónoma de Madrid, 28049 Madrid, Spain; 9 Servicio de Neumología, Clínica Universidad de Navarra, 28027 Madrid, Spain; 10 Fundació Institut d’Investigació i Innovació Parc Taulí (I3PT), 08208 Sabadell, Spain; 11 Servicio de Neumología, Hospital Son Espases, Institut d’Investigació Sanitària Illes Balears (IdISBa), Universitat de les Illes Balears, 07120 Palma, Spain

## Error in Table 4

In the original publication, the authors identified a mistake in Table 4 as published [1]: the values in Table 4 were duplicated from Table 3. The corrected Table 4 appears below. The authors state that the scientific conclusions are unaffected. This correction was approved by the Academic Editor. The original publication has also been updated.

## Figures and Tables

**Table 4:** Comparison of prediction models using conventional DAMs versus AI-based free metabolite selection combined with FEV_1_ integration.

	Conventional + FEV_1_	AI Free Choice + FEV_1_
**Fitting**		
Sensitivity–Specificity–MCC	1.00	1.00
Coverage	1.00	1.00
**Prediction**		
Sensitivity	0.83	1.00
Specificity	1.00	0.92
MCC	0.83	0.92
Coverage	0.85	0.96
PPV (rep/our)	1.00/1.00	0.92/0.86
NPV (rep/our)	0.87/0.93	1.00/1.00
Accuracy (rep/our)	0.92/0.95	0.96/0.95

PPV: NPV and accuracy were calculated based on either (a) the mortality observed in our COPD cohort (32%) [our] or (b) the mortality rate previously reported [rep] in the Spanish COPD population (47%) [11], which aligns with mortality predictions derived from the 3CIA initiative detailed in Appendix B [6,12]. Abbreviations: MCC: Matthews correlation coefficient; PPV: positive predicted value; NPV: negative predicted value; rep: reported mortality; our: our own mortality; AI: artificial intelligence.

## Appendix A

Members of the BIOMEPOC group (in alphabetical order): Mireia Admetlló, Alvar Agustí, Carlos Alvarez-Martínez, Esther Barreiro, Oswaldo Antonio Caguana, Carme Casadevall, Ferran Casals, Robert Castelo, Ady Castro-Acosta, Rocío Córdova, Borja G Cosío, Rosa Faner, Laura I Furlong, Marian García, José G. González-García, Carmen Hernández-Carcereny, José Luis López-Campos, Eduardo Márquez, Eduard Monsó, Concepción Montón, Miren Josune Ormaza, Alexandre Palou, Sergi Pascual, Germán Peces-Barba, Pau Puigdevall, Diego A. Rodríguez-Chiaradia, Ferran Sanz, Luis Seijo, Montserrat Torà, Yolanda Torralba, and Carles Vilaplana.
