# Supplementary material for: Metabolomic Signatures Predict Seven-Year Mortality in Clinically Stable COPD Patients
Source: Int J Mol Sci. 2025 Jul 2;26(13):6373. doi: 10.3390/ijms26136373 (PMC12249577; doi:10.3390/ijms26136373)
Supplement: Supplementary file 1 [file ijms-26-06373-s001.zip › ijms-3702454-supplementary.pdf]

Table S1. Differences in metabolite levels between deceased and surviving COPD patients after 7 years of follow-up, adjusted for age and post-bronchodilator FEV_1.

Metabolite	logFC	%Δ	P.Value	Type
Butyryl-L-carnitine	0,8233	76,95	0,0028043	endogen
Levodopa	1,1807	126,69	0,0056229	endogen
Hydroxyproline	0,6793	60,14	0,0087264	endogen
IndoxylSulfate	1,0562	107,94	0,0113590	mix End-Micro
L-Cystine	-1,0506	-51,72	0,0207147	endogen
Phenylac-Gln-OH	1,0313	104,38	0,0247220	mix End-Micro
1-Methylnicotinamide	-0,7350	-39,92	0,0250523	endogen
4-Aminobenzoate	-0,8362	-43,99	0,0324650	microbioma
Trigonelline	-0,8248	-43,54	0,0333086	Xeno
Monomethyl glutarate	0,8077	75,05	0,0349329	endogen
Thiopurine S-Methylether	0,9466	92,73	0,0455578	Xeno
Citric acid	-1,0226	-50,78	0,0465788	endogen
Creatine	0,5618	47,61	0,0486943	endogen
Pyruvic acid	0,2938	22,58	0,0534305	endogen
2-Hydroxyglutaric acid	-0,6872	-37,89	0,0558631	endogen
Benzyl Alcohol	0,9308	90,64	0,0596445	Xeno
cyclo-Dopa	-0,7407	-40,16	0,0638037	Xeno
Proline	0,3449	27,01	0,0690995	endogen
Phenol	1,2227	133,39	0,0729924	microbioma
3-Methyl-2-oxovaleric acid	0,5672	48,17	0,0770763	endogen
Acetyl-beta-methylcholine	0,4969	41,12	0,0799983	Xeno
2-Phosphoglyceric acid	0,5983	51,39	0,0841422	endogen
Aspartate	0,4243	34,19	0,0907333	endogen
2-Deoxy-D-Glucose	-0,4271	-25,62	0,0932061	Xeno
Threitol	0,5532	46,73	0,0940024	microbioma
Urocanate	-0,3272	-20,29	0,1006106	endogen
2-Hydroxypyridine	-0,4948	-29,03	0,1010628	Xeno
beta-alanine	-0,4145	-24,97	0,1029852	endogen
4-Hydroxybenzoate	1,1598	123,43	0,1031404	microbioma
Citramalate	-0,5934	-33,72	0,1078906	microbioma
2-Keto-glutaramic acid	-0,3444	-21,23	0,1119049	endogen
Hydroquinone	-0,4919	-28,89	0,1212496	Xeno
Propionylcarnitine	0,3779	29,95	0,1280898	endogen
Rhamnose	-0,5021	-29,39	0,1309881	microbioma
Theophylline	-1,5355	-65,50	0,1344844	Xeno
Tetraglyme	0,3614	28,47	0,1350492	Xeno
Octanoic acid	0,5251	43,91	0,1350563	endogen
Itaconate	-0,6789	-37,53	0,1384935	endogen
Pentapropylene glycol	0,4393	35,59	0,1387765	Xeno
Pentanoate	0,5107	42,47	0,1405032	endogen
Myristoylcarnitine	0,3259	25,34	0,1425846	endogen
Octanoyl-carnitine	0,5605	47,48	0,1646502	endogen
Kynurenine	-0,2832	-17,82	0,1660566	endogen
Decanoylcarnitine	0,4615	37,70	0,1674375	endogen
Omeprazole	1,4935	181,58	0,1746615	Xeno
N-Acetyltryptophan	0,2907	22,32	0,1755294	endogen
allophanic acid	0,3031	23,38	0,1816342	Xeno
5-Hydroxyindoleacetate	-0,2723	-17,20	0,1836860	mix End-Micro
DL-Dipalmitoylphosphatidylcholine	0,2184	16,34	0,1905209	endogen

3-Methoxytyrosine	-0,5534	-31,86	0,1921762	endogen
Hexanoyl-L-carnitine	0,5262	44,01	0,1984578	endogen
Orotate	0,3799	30,12	0,2039982	endogen
Choline	-0,2514	-15,99	0,2087069	endogen
2-Methylcitrate	0,2780	21,25	0,2165719	endogen
Mannitol	-0,7355	-39,94	0,2212503	microbioma
Cytidine	-0,4027	-24,36	0,2342565	endogen
Cotinine	1,2050	130,53	0,2354539	Xeno
(5Z,8Z,11Z)-Eicosatrienoic acid	0,3289	25,60	0,2388952	endogen
Nonadecanoic acid	-0,2137	-13,77	0,2398210	endogen
Lactate	0,2409	18,18	0,2421901	endogen
(S)-(+)-2-Amino-1-propanol	0,4148	33,31	0,2464968	Xeno
Argininosuccinate	-0,3714	-22,70	0,2477318	endogen
N-Carbobenzoxy-beta-alanine	-0,2066	-13,34	0,2539725	Xeno
2,3-Dihydroxybenzoic acid	-0,6026	-34,15	0,2655857	microbioma
Pyridoxal	0,2981	22,95	0,2666576	endogen
Hypotaurine	0,2638	20,06	0,2756193	endogen
cyclo-Dopa 5-O-glucoside	0,3144	24,35	0,2768342	endogen
Serotonin	0,5092	42,33	0,2769026	endogen
Maybridge3_001052	-0,1886	-12,25	0,2811381	Xeno
Monoethyl malonate	-0,2630	-16,67	0,2814432	unclassifiable
TDIQ	0,2621	19,92	0,2817856	Xeno
Arachidonic acid	0,2525	19,13	0,2836482	endogen
2-(2-Carboxyethyl)-4-methyl-5-propylfuran-3-carboxylicacid	-0,5314	-30,81	0,2876854	microbioma
beta-Hydroxyisovaleric acid	0,4010	32,04	0,3007065	endogen
Deoxyguanosine monophosphate	0,7283	65,67	0,3069023	endogen
Betaine	-0,2708	-17,12	0,3075713	endogen
3-amino-2,3-dideoxy-scyllo-inosose	0,1924	14,26	0,3087907	microbioma
5-Nitro-o-toluidine	-0,4162	-25,06	0,3096074	Xeno
Leucylproline	-0,2126	-13,70	0,3100325	endogen
MyoInositol	0,3199	24,83	0,3121166	endogen
Palmitoylsphingomyelin	0,1855	13,72	0,3137728	endogen
Sarcosine	0,1852	13,70	0,3157088	endogen
Thyroxine	0,2301	17,30	0,3201086	endogen
Decanoic acid	0,2183	16,34	0,3254721	endogen
Didecyldimethylammonium	0,6461	56,50	0,3278030	Xeno
9-HpODE	-0,2661	-16,84	0,3328278	endogen
Propionic acid	0,1782	13,15	0,3341879	microbioma
Ecgonine	-0,2050	-13,24	0,3367439	Xeno
D-Mannosamine	-0,4715	-27,88	0,3388450	endogen
Trans-3-Indoleacrylic acid	-0,5905	-33,59	0,3441160	microbioma
Lumichrome	-0,2150	-13,85	0,3576531	microbioma
Alanine	0,1585	11,61	0,3588070	endogen
MethylGalactoside	-0,5054	-29,55	0,3590391	unclassifiable
Glycochenodeoxycholate	0,9506	93,27	0,3606174	mix End-Micro
Stearidonic acid	-0,5302	-30,75	0,3613014	endogen
Mevalonate	-0,2420	-15,44	0,3646122	endogen
Oxalate	0,1502	10,97	0,3739190	endogen
Minoxidil	-0,4711	-27,86	0,3768414	Xeno
Dihydrothymine	-0,3469	-21,37	0,3769673	endogen
Tartrate	0,7172	64,40	0,3819383	endogen
N-Acetylphenylalanine	-0,2082	-13,44	0,3893095	endogen

Hexaethylene glycol	-0,1617	-10,60	0,3981871	Xeno
N-Acetylleucine	0,1860	13,76	0,4066030	endogen
S-Adenosylhomocysteine	0,2085	15,55	0,4110757	endogen
Sphinganine	0,2253	16,91	0,4117811	endogen
Adrenic acid	0,3201	24,84	0,4123656	endogen
1-Tetradecylamine	-1,1632	-55,35	0,4125361	unclassifiable
Valine	0,1407	10,25	0,4163442	endogen
Azelate	-0,1937	-12,56	0,4199784	microbioma
D-Ribose 5-phosphate	0,1817	13,42	0,4336304	endogen
(S)-methyl-2-acetamido-4-(2-(methylamino)phenyl)-4-oxobutanoate	0,1437	10,47	0,4344648	endogen
Tyrosine	-0,1424	-9,40	0,4468729	endogen
Cystathionine	0,2689	20,49	0,4473690	endogen
2-(3,4-dihydroxyphenyl)-3,5,7-trihydroxy-6-methyl-4H-chromen-4-one	0,1195	8,64	0,4481901	Xeno
Cytosine	-0,2393	-15,28	0,4499371	endogen
Inosine monophosphate	0,1080	7,78	0,4526864	endogen
L-Cysteic acid	-0,3323	-20,57	0,4533073	endogen
Acetaminophen glucuronide	0,2629	19,99	0,4548614	Xeno
cis-5-Dodecenoic acid	0,2076	15,48	0,4576053	microbioma
N-Acetylserine	0,1282	9,30	0,4615705	endogen
Isoprene	0,1495	10,92	0,4620527	endogen
L-Isoleucine	0,1437	10,47	0,4660459	endogen
N-Acetylneuraminate	0,1328	9,64	0,4666030	endogen
N-Nitrosodiethylamine	-0,1018	-6,81	0,4710223	Xeno
L-Histidine	-0,0988	-6,62	0,4809091	endogen
N-Formylglycine	0,1385	10,08	0,4831929	endogen
n-6 docosapentaenoate	0,2506	18,97	0,4836675	endogen
Lauroylcarnitine	0,1651	12,12	0,4838123	endogen
4-Guanidinobutanoic acid	-0,2266	-14,53	0,4890442	endogen
Adenine	-0,1261	-8,37	0,4917629	endogen
Palmitoylcarnitine	0,1037	7,45	0,4917726	endogen
Guvacoline	0,2521	19,09	0,4955160	Xeno
2,4-Diaminopentanoic acid	-0,1621	-10,63	0,4988786	endogen
Threonine	0,1167	8,43	0,4992883	endogen
cis-10-Nonadecenoic acid	0,3388	26,47	0,5012318	endogen
Allantoin	-0,1636	-10,72	0,5019121	endogen
2-Aminoadipic acid	0,1744	12,85	0,5022083	endogen
acetoacetic acid	0,1194	8,63	0,5039409	Xeno
Allothreonine	0,1159	8,36	0,5042496	endogen
1-Aminocyclopropanecarboxylate	-0,1239	-8,23	0,5070710	endogen
Gulonolactone	-0,1231	-8,18	0,5079047	endogen
Urate	-0,1217	-8,09	0,5088702	endogen
5-Valerolactone	0,0952	6,82	0,5089047	endogen
Diethanolamine	-0,1300	-8,62	0,5092456	Xeno
Behenic acid	-0,1658	-10,86	0,5122461	endogen
Homoserine	0,1130	8,15	0,5132901	endogen
Mevalolactone	-0,2406	-15,36	0,5145641	endogen
6-Methyl[1,2,4]triazolo[4,3-b]pyridazin-8-ol	0,1305	9,47	0,5157791	Xeno
Acetylarginine	-0,1561	-10,26	0,5192060	endogen
9-Decenoic acid	0,1845	13,64	0,5219350	microbioma
Palmitoleic acid	0,2822	21,60	0,5222550	endogen

Linoleic acid	0,2431	18,36	0,5237365	endogen
1-Aminocyclopropane-1-carboxylic acid	0,1099	7,91	0,5250777	Xeno
Docosahexaenoic Acid	0,1461	10,65	0,5353508	endogen
Cinnamate	-0,0979	-6,56	0,5370388	microbioma
Phenacetin	0,1075	7,74	0,5373020	Xeno
Gluconic acid	0,1299	9,42	0,5379973	Xeno
Guanine	-0,4148	-24,99	0,5420853	endogen
Nicotinamide	-0,1590	-10,44	0,5441964	endogen
Pentadecanoic acid	-0,1320	-8,75	0,5510881	endogen
Tryptophan	-0,1072	-7,16	0,5538740	endogen
Ethylenediaminetetraacetic acid (EDTA)	0,0968	6,94	0,5579900	Xeno
Caffeine	-0,2976	-18,64	0,5584957	Xeno
Prolylleucine	-0,1161	-7,73	0,5589900	endogen
3-(4-Hydroxyphenyl)pyruvic acid	-0,1304	-8,64	0,5634645	endogen
Biotin	-0,1470	-9,69	0,5634972	endogen
Citrate	0,0917	6,57	0,5676946	endogen
DL-Histidine	-0,4624	-27,42	0,5690324	endogen
4-Imidazoleacetic acid	0,1746	12,86	0,5692278	endogen
Methylarginine	-0,1466	-9,66	0,5714082	endogen
alphaKetoglutarate	-0,1059	-7,07	0,5728673	endogen
Glycolate	0,0829	5,91	0,5729168	endogen
N3-Phenyl-1H-1,2,4-triazole-3,5-diamine	0,0908	6,49	0,5733169	Xeno
Dehydroascorbate	-0,3254	-20,19	0,5742138	endogen
Quinate	-0,3157	-19,65	0,5769909	microbioma
Citrulline	-0,1272	-8,44	0,5775684	endogen
Hippurate	0,2814	21,54	0,5781068	mix End-Micro
Phenylalanine	-0,0850	-5,72	0,5783276	endogen
Guanidinoacetate	-0,1177	-7,84	0,5808200	endogen
N6-Methyl-L-lysine	0,2467	18,65	0,5832023	endogen
2-Naphthalenesulfonic acid	0,1680	12,35	0,5880847	Xeno
Methyl Vanillate	-0,1248	-8,29	0,5886033	Xeno
8-Hydroxyquinoline	-0,0962	-6,45	0,5895909	Xeno
2-Oxa-4-azatetracyclo[6.3.1.16,10.01,5]tridecane	-0,2533	-16,10	0,5897811	Xeno
Xanthine	-0,1534	-10,09	0,5909737	endogen
Leucine	0,1046	7,52	0,5916245	endogen
Dimethylglycine	0,1299	9,42	0,5936397	endogen
Glycerol-3-Phosphate	-0,3935	-23,87	0,5941775	endogen
Trehalose	0,2109	15,74	0,5998869	microbioma
Glycocholate	-0,3146	-19,59	0,6023921	mix End-Micro
Benzaldehyde	-0,0946	-6,35	0,6043179	microbioma
Anserine	-0,4625	-27,43	0,6043191	endogen
Arginine	-0,1134	-7,56	0,6050298	endogen
Bis(2-Ethylhexyl) Phthalate	-0,2181	-14,03	0,6077003	Xeno
Galactarate	0,1005	7,21	0,6118227	microbioma
Levulinic acid	0,0775	5,52	0,6146801	Xeno
5-Methylcytosine	-0,1085	-7,25	0,6154485	endogen
3-Hydroxybutanoate	0,1561	11,43	0,6218543	endogen
N(6)-(delta(2)-Isopentenyl)adenine	0,0856	6,11	0,6222487	unclassifiable
N-Acetylornithine	0,1919	14,23	0,6279937	endogen
Phosphocreatine	0,1207	8,73	0,6283429	endogen
Dethiobiotin	-0,1098	-7,33	0,6339336	endogen

Asparagine	-0,0722	-4,88	0,6368317	endogen
2-Aminonicotinic acid	-0,0763	-5,15	0,6380629	endogen
Guanidinosuccinate	-0,1863	-12,11	0,6414086	endogen
4-Coumarate	-0,1273	-8,45	0,6482814	microbioma
Deoxycholate	0,2295	17,24	0,6498644	endogen
4,4'-dimethoxy[1,1'-biphenyl]-2-carbonitrile	0,1275	9,24	0,6571296	Xeno
N?-Acetyl-L-lysine	-0,0914	-6,14	0,6639211	endogen
5-Methylthio-D-ribose	0,0726	5,16	0,6832541	endogen
Inosine	-0,2404	-15,35	0,6832784	endogen
DL-Glyceric acid	0,1888	13,98	0,6854226	endogen
4-Acetamidobutanoic acid	0,1292	9,37	0,6856032	endogen
Glutarate	0,0652	4,63	0,6890187	endogen
Glucose	0,0728	5,18	0,6899802	endogen
alpha-Hydroxyisobutyric acid	-0,2075	-13,40	0,6934170	endogen
3-Hydroxybutyric acid	0,1152	8,31	0,6943960	endogen
Lactose	0,1226	8,87	0,6953676	microbioma
N-Acetyl-L-alanine	0,0617	4,37	0,6961838	endogen
NAcetylglycine	-0,0718	-4,86	0,7010845	endogen
Kynurenate	-0,2639	-16,71	0,7045870	endogen
Methylthioadenosine	-0,0759	-5,13	0,7057978	endogen
Adenosine	-0,1250	-8,30	0,7064839	endogen
Pipecolate	0,1465	10,69	0,7174307	mix End-Micro
S-Cysteinosuccinic acid	0,0800	5,70	0,7218497	endogen
Maleate	-0,0823	-5,54	0,7222193	endogen
Bilirubin	-0,1197	-7,96	0,7239627	endogen
Acetaminophen	0,1231	8,91	0,7311810	Xeno
Oleic acid	0,1140	8,22	0,7347957	endogen
2-Hydroxyhexadecanoic acid	-0,0418	-2,86	0,7365009	endogen
Serine	0,0614	4,35	0,7394390	endogen
3-Methylglutaconate	-0,0693	-4,69	0,7403855	endogen
3-Hydroxymethylglutarate	0,0582	4,12	0,7411393	endogen
1,5-Naphthalenediamine	-0,0598	-4,06	0,7415026	Xeno
4-Hydroxybenzaldehyde	-0,1045	-6,99	0,7418442	microbioma
Prolinamide	-0,0559	-3,80	0,7427431	unclassifiable
cis-Aconitate	0,0524	3,70	0,7428782	endogen
Creatinine	-0,0560	-3,81	0,7458784	endogen
Methionine	-0,0569	-3,86	0,7480583	endogen
Cyclic AMP	-0,0569	-3,87	0,7508561	endogen
3-Hydroxybenzaldehyde	-0,1001	-6,70	0,7523716	microbioma
Didemethylisoproturon	-0,1085	-7,25	0,7526713	Xeno
1-Methyladenosine	0,0393	2,76	0,7619838	endogen
Ornithine	-0,0577	-3,92	0,7680005	endogen
Myristoleic acid	0,0965	6,92	0,7702080	endogen
Adenosine Phosphate	-0,1046	-7,00	0,7710447	endogen
L-Pipecolic acid	0,1785	13,17	0,7713506	endogen
N-Acetylasparagine	0,0672	4,77	0,7749339	endogen
Triethanolamine	-0,0701	-4,74	0,7754080	Xeno
Galactosamine	0,0488	3,44	0,7761081	endogen
Xylitol	-0,0832	-5,60	0,7761093	microbioma
L-Methionine sulfoxide	0,0587	4,16	0,7793293	endogen
Butyric acid	-0,0433	-2,96	0,7804606	microbioma
2-Methylmaleic acid	-0,0491	-3,35	0,7834566	endogen

Dihydrobiopterin	-0,0832	-5,60	0,7853905	endogen
Fumarate	-0,0533	-3,63	0,7858206	endogen
Tridecanoic acid	-0,0412	-2,82	0,7975558	endogen
Uridine diphosphate glucose	-0,1522	-10,01	0,7980321	endogen
Malate	-0,0513	-3,50	0,8009559	endogen
Pentaethylene glycol	0,0354	2,49	0,8038434	Xeno
Glycolic acid	0,0411	2,89	0,8061637	endogen
N-Methylglutamate	0,0406	2,85	0,8065884	microbioma
Stachydrine	-0,2060	-13,30	0,8066602	unclassifiable
Succinate	0,0326	2,29	0,8076812	endogen
Glutamate	-0,0654	-4,43	0,8087336	endogen
Pyruvate	-0,0443	-3,02	0,8122855	endogen
Raffinose	0,0701	4,98	0,8123822	microbioma
2-Hydroxytetradecanoic acid	-0,0513	-3,49	0,8171417	endogen
5-Hydroxylysine	0,0709	5,04	0,8258955	endogen
Tricosanoic acid	-0,0694	-4,70	0,8282806	endogen
3-Nitro-L-tyrosine	-0,0420	-2,87	0,8302915	endogen
cis-11-Eicosenoic acid	0,0668	4,74	0,8306561	endogen
Glutamine	-0,0292	-2,01	0,8311885	endogen
N-Methyl-2-pyrrolidone	0,1444	10,53	0,8319461	Xeno
Indole-3-acetic acid	0,0834	5,95	0,8336252	microbioma
Lotaustralin	0,0382	2,69	0,8342872	Xeno
N-Acetylputrescine	0,0435	3,06	0,8363707	endogen
PPG n4	-0,0305	-2,09	0,8380132	Xeno
Sucrose	0,0729	5,18	0,8391699	microbioma
Nonanoic acid	-0,0437	-2,99	0,8397979	endogen
N-Acetylglutamate	-0,0458	-3,12	0,8432704	endogen
2-Aminophenol	0,0647	4,59	0,8454683	Xeno
Myristic acid	-0,0324	-2,22	0,8461566	endogen
D-Glucosamine 6-phosphate	0,0640	4,54	0,8480269	endogen
L-Threonic acid	0,0532	3,75	0,8480965	endogen
Diethyl 2-Methyl-3-Oxosuccinate	-0,0536	-3,65	0,8483560	Xeno
Taurine	0,0366	2,57	0,8493013	endogen
Glutarylcarnitine	-0,0542	-3,69	0,8541527	endogen
Thymine	-0,0275	-1,89	0,8546232	endogen
Arabic acid	-0,0598	-4,06	0,8551761	microbioma
Lignoceric acid	-0,0409	-2,80	0,8581804	endogen
Oxoproline	-0,0255	-1,75	0,8623721	endogen
Pantothenate	-0,0345	-2,36	0,8668390	endogen
Stearic acid	0,0136	0,94	0,8670326	endogen
N,N-Diisopropylethylamine	-0,0889	-5,98	0,8678038	Xeno
3-Hydroxyoctanoic acid	-0,1557	-10,23	0,8692189	endogen
Ricinoleic acid	-0,0514	-3,50	0,8763184	endogen
Pseudouridine	0,0276	1,93	0,8795664	endogen
Biliverdin	0,1228	8,89	0,8798629	endogen
4-Dodecylbenzenesulfonic acid	-0,0377	-2,58	0,8812051	Xeno
Palmitate	-0,0089	-0,61	0,8830474	endogen
3-Methoxy-4-hydroxymandelic acid	0,0650	4,61	0,8911975	microbioma
DL-2,6-Diaminopimelic acid	-0,0261	-1,80	0,8921723	microbioma
Quinolinate	0,0512	3,61	0,8977326	endogen
Glycerol	-0,0319	-2,19	0,8999015	endogen
Indole-3-Pyruvate	-0,0297	-2,04	0,9003859	microbioma
N-Acetyl-L-glutamine	-0,0191	-1,32	0,9014112	endogen

Aminoisobutanoate	0,0354	2,49	0,9025381	endogen
Arachidic acid	0,0101	0,70	0,9075605	endogen
Lauric acid	-0,0291	-2,00	0,9077587	endogen
Acetoacetate	-0,0269	-1,85	0,9105148	endogen
Dimethylarginine	-0,0157	-1,08	0,9119655	endogen
Eicosapentaenoate	-0,0470	-3,21	0,9134503	endogen
Cholate	-0,0754	-5,09	0,9173838	endogen
Linolenic acid	-0,0353	-2,42	0,9186385	endogen
Ximeninic Acid	-0,0353	-2,42	0,9186385	endogen
Hypoxanthine	-0,0319	-2,18	0,9192843	endogen
Trimethyllysine	0,0250	1,75	0,9211094	endogen
3-Methyl-L-Histidine	-0,0295	-2,02	0,9217068	endogen
Phosphorylcholine	0,0198	-1,14	0,9319606	endogen
Acetyl-L-carnitine	-0,0162	1,38	0,9364470	endogen
Quinoline	-0,0309	-1,12	0,9379399	Xeno
Methylimidazoleacetic acid	-0,0295	-2,12	0,9383888	Xeno
D-Erythro-sphingosine 1-phosphate	0,0139	-2,03	0,9389993	endogen
D-Proline	0,0120	0,97	0,9403682	endogen
Deoxyribose	0,0241	0,84	0,9455264	endogen
Carnitine	0,0116	1,69	0,9478704	endogen
14-Methylhexadecanoic acid	-0,0073	0,81	0,9495832	endogen
Sn-Glycero-3-phosphocholine	-0,0124	-0,50	0,9501052	endogen
beta-Glycerophosphoric acid	0,0122	-0,86	0,9511503	endogen
Uridine	0,0091	0,85	0,9553898	endogen
Homocysteine	0,0119	0,63	0,9606924	endogen
Bis(methylbenzylidene)sorbitol	-0,0034	0,83	0,9763624	Xeno
Deoxycarnitine	0,0061	-0,24	0,9780639	endogen
Xylose	0,0049	0,42	0,9791660	microbioma
Ribose	0,0042	0,34	0,9820872	endogen
Glycine	0,0026	0,29	0,9856132	endogen
Uracil	0,0027	0,18	0,9861006	endogen
Lysine	-0,0027	0,19	0,9881965	endogen
Phosphoethanolamine	0,0044	-0,18	0,9888619	endogen
Succinyladenosine	-0,0025	0,30	0,9900276	endogen
1,3,4-Trihydroxy-5-oxocyclohexanecarboxylic acid	-0,0027	-0,18	0,9907706	endogen

Table S2. *Post-hoc* analysis of metabolite differences between deceased and surviving COPD patients after 7 years of follow-up, excluding early deaths (<2 years), adjusted for age and post-bronchodilator FEV_1.

Metabolite	logFC	%Δ	P.Value	Type
Butyryl-L-carnitine	0.7469	67.82	0.01	endogen
Levodopa	1.2017	130.00	0.02	endogen
Hydroxyproline	0.8536	80.71	0.00	endogen
IndoxylSulfate	0.7854	72.36	0.07	mix End-Micro
L-Cystine	-1.0572	-51.94	0.02	endogen
Phenylac-Gln-OH	0.7566	68.94	0.12	mix End-Micro
1-Methylnicotinamide	-0.8541	-44.68	0.01	endogen
4-Aminobenzoate	-0.2414	-15.41	0.52	microbioma
Monomethyl glutarate	0.7649	69.92	0.07	endogen
Citric acid	-0.8087	-42.91	0.10	endogen
Creatine	0.5967	51.23	0.06	endogen
Pyruvic acid	0.2829	21.67	0.09	endogen
2-Hydroxyglutaric acid	-0.6276	-35.27	0.09	endogen
Proline	0.3298	25.69	0.13	endogen
Phenol	0.9201	89.23	0.22	microbioma
3-Methyl-2-oxovaleric acid	0.4862	40.07	0.18	endogen
2-Phosphoglyceric acid	0.5312	44.51	0.16	endogen
Aspartate	0.4807	39.54	0.08	endogen
Threitol	0.5306	44.46	0.15	microbioma

Figure S1. Receiver operating characteristic (ROC) curves for mortality prediction models

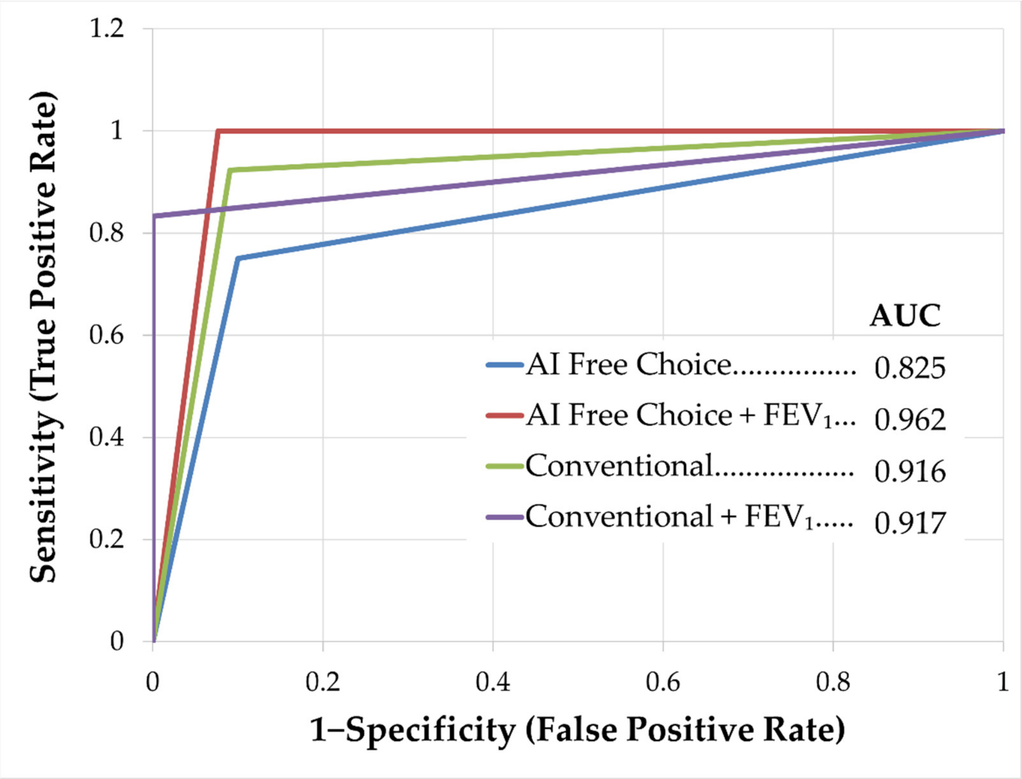

ROC curves comparing the performance of four Random Forest classification models for 7-year mortality prediction in COPD patients. "Conventional" models were built using the top 10 differentially abundant metabolites (DAMs), whereas "AI Free Choice" models allowed the algorithm to select the most informative features using K-best selection. Each model was tested with and without the inclusion of post-bronchodilator FEV_1 as an additional input variable. The area under the curve (AUC) is reported for each model.
